# The American Agriculturist

**Published:** 1873-12

**Authors:** 


*The American Agriculturist.* Published by Orange, Judd & Co. 545 Broadway, New York, at $1 50 per annum.

This journal the past year fully sustains the character it has so long borne of being one of the best publications of the kind issued. No labor or expense is spared to render its pages instructive as well as entertaining to all engaged in agricultural pursuits. It is issued monthly, and the twelve numbers when bound make a handsome volume, so liberally are they illustrated.

